# Individual Influence in Population Pharmacokinetics Depends on Underlying Mechanism: Evidence from a Jackknife ΔOFV Approach

**DOI:** 10.3390/pharmaceutics18080984

**Published:** 2026-08-10

**Authors:** Nicolas Simon, Katharina von Fabeck

**Affiliations:** Institut de Neurosciences de la Timone, UMR7289 CNRS, Hop Sainte Marguerite, Department of Clinical Pharmacology, Centre Anti-Poison Toxicovigilance (CAP), Aix-Marseille University, APHM, 13005 Marseille, France; katharina.moenikes@ap-hm.fr

**Keywords:** pharmacometry, population pharmacokinetics, jackknife, influence analysis, NONMEM, simulation study, model diagnostics

## Abstract

**Background**: Identifying influential individuals is a critical step in nonlinear mixed-effects modeling, yet commonly used diagnostics primarily reflect local model fit or parameter perturbation and may fail to capture the full impact of individual data on model estimation. **Methods**: We conducted a simulation study based on a one-compartment pharmacokinetic model with first-order absorption. Four scenarios were investigated: a reference scenario without induced influence, a residual outlier scenario, a structurally influential individual with enriched sampling, and a latent subpopulation scenario. For each scenario, 25 datasets of 100 individuals were simulated, and individual influence was assessed using a leave-one-out jackknife approach. Influence metrics included the change in objective function value (ΔOFV) and parameter perturbation measures. Detection performance was evaluated at both subject and dataset levels. **Results**: All jackknife runs were successfully completed and analyzable. Residual outliers were consistently identified by both ΔOFV and parameter-based metrics. In contrast, structurally influential individuals were reliably detected by ΔOFV (median rank = 1) but not by parameter-based metrics (median rank ≈ 25). Across scenarios, the association between ΔOFV and parameter perturbation was weak, and nearly absent in the structurally influential scenario. In the latent subpopulation scenario, individual-level detection was limited, but dataset-level detection remained effective, with at least one subpopulation member frequently identified among top-ranked individuals. **Conclusions**: Individual influence in nonlinear mixed-effects models is strongly mechanism-dependent. Jackknife-based ΔOFV provides a direct and general measure of individual influence, capable of detecting both residual outliers and structurally influential individuals. In contrast, parameter-based metrics quantify parameter sensitivity rather than individual influence and may therefore overlook influential subjects in specific contexts. These findings support the use of jackknife deletion as a reference approach for influence assessment in pharmacometric workflows.

## 1. Introduction

Population pharmacokinetic modelling using nonlinear mixed-effects models (NLME) has become a standard framework in clinical pharmacology for characterising drug exposure variability and informing dosing strategies in heterogeneous patient populations [1,2]. A fundamental assumption underlying these analyses is that population parameter estimates are robust to individual subjects in the dataset. In practice, certain individuals may exert disproportionate influence on fixed-effect estimates, whether as statistical outliers, high-leverage subjects, or members of an unmodelled subpopulation, potentially biasing the model and compromising its generalisability [3,4].

Several diagnostic approaches are routinely used to detect model misspecification and atypical individuals in pharmacometric practice. Conditional weighted residuals (CWRES) provide a subject-level measure of local misfit [5], while individual contributions to the objective function value (iOFV) offer a likelihood-based indicator of how well the model accounts for each subject’s data [6]. These tools are widely available and computationally inexpensive, but their ability to capture the true impact of an individual on global parameter estimation has not been formally characterised. A key limitation is that both metrics are derived from the full model fit where, as conditional individual estimates, they are further affected by shrinkage [7] and do not directly quantify what would change if a given subject were removed.

The leave-one-out jackknife addresses this limitation directly: by iteratively removing each subject and re-estimating the model, it yields changes in objective function value (ΔOFV) and parameter estimates (Δθ) that reflect individual influence in an unambiguous, model-agnostic manner [8,9]. We focus on these two jackknife-derived quantities because they capture complementary aspects of individual influence that are directly relevant to pharmacometric inference: ΔOFV measures the global impact of a subject on the model likelihood, whereas Δθ measures the sensitivity of the fixed-effect parameter estimates that ultimately drive exposure and dosing predictions. Characterising when these two quantities agree or diverge is therefore central to the reliable interpretation of population parameter estimates. Jackknife deletion has been applied in population pharmacokinetics to assess model stability and to identify subjects influencing covariate selection [3,4], but the extent to which its two derived metrics, ΔOFV and Δθ, capture distinct mechanisms of individual influence has not been systematically characterised under controlled simulation conditions. Furthermore, its practical adoption remains limited by dependence on PsN, a Perl-based infrastructure that requires Unix-compatible environments and presents compatibility barriers in many clinical and academic settings [10].

Approximate case-deletion methods such as linearized dOFV have been proposed to reduce computational burden [11], but their ability to capture all mechanisms of influence remains uncertain.

In this study, we present a fully automated, R-based pipeline for leave-one-out jackknife analysis in NONMEM, independent of PsN and compatible with standard Windows environments, the primary methodological contribution of this work. Using a simulation study, we compared two jackknife-derived metrics, ΔOFV and Δθ, across four mechanistically distinct scenarios: a reference condition without contamination, residual outliers, structurally influential subjects with increased informational content, and latent subpopulations. The aim was to characterise the conditions under which each approach reliably identifies influential individuals and to propose a practical workflow for routine pharmacometric practice.

## 2. Methods

### 2.1. Study Design

**Figure 1:**
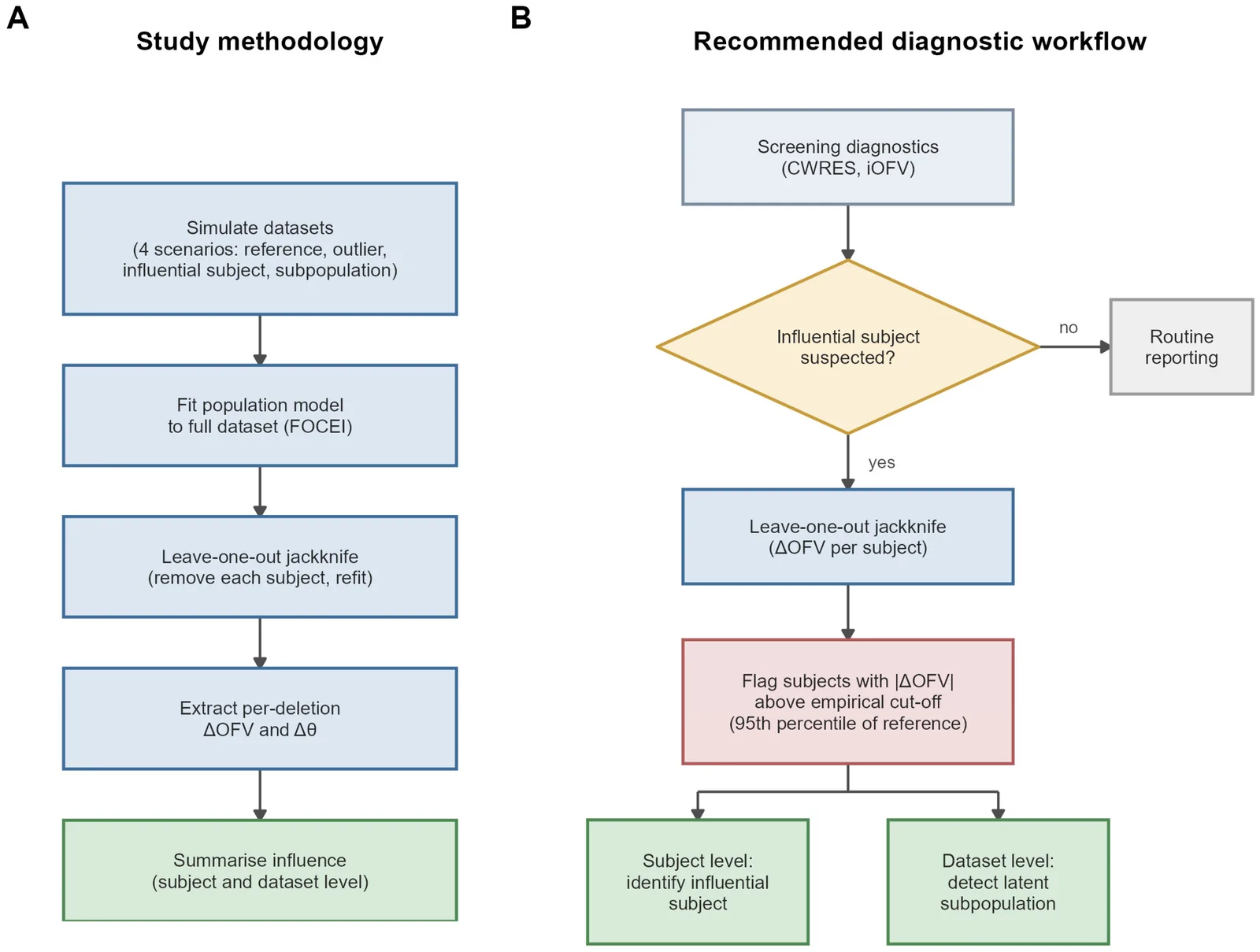
Overview of the study methodology and the recommended diagnostic workflow. (**A**) Study methodology. For each of the four scenarios (reference, residual outlier, structurally influential subject, latent subpopulation), datasets are simulated from the one-compartment model; a population model is fitted to the full dataset by FOCEI; each subject is then removed in turn, and the model refitted (leave-one-out jackknife); the per-deletion change in objective function value (ΔOFV) and in the fixed-effect parameters (Δθ) is extracted; and individual influence is summarised and interpreted at both the subject and dataset levels. (**B**) Recommended diagnostic workflow. Low-cost screening diagnostics (CWRES, iOFV) are applied first; when an influential subject is suspected, the leave-one-out jackknife is run and subjects are flagged when their |ΔOFV| exceeds an empirical cut-off (the 95th percentile of the reference-scenario |ΔOFV| distribution); influence is then interpreted at the subject level (identification of an influential individual) and at the dataset level (detection of a latent subpopulation).

A simulation-based pharmacometric study was conducted to evaluate the influence of individual subjects on population parameter estimates using a jackknife approach applied to nonlinear mixed-effects models. An overview of the simulation workflow and the recommended diagnostic strategy is provided in Figure 1.

### 2.2. Data Simulation

Datasets were simulated using a one-compartment pharmacokinetic model with first-order absorption. The typical values for clearance (CL), volume of distribution (V), and absorption rate constant (KA) were set to 5 L/h, 50 L, and 1 h^−1^, respectively. Inter-individual variability was introduced on CL and V using exponential models with normally distributed random effects (mean 0, standard deviation 0.3).

Each dataset included 100 individuals, each with 8 sampling times (0, 0.5, 1, 2, 4, 8, 12, and 24 h). Drug administration consisted of a single oral dose of 100 units at time 0. Observations were simulated using the analytical solution of the one-compartment model, with proportional residual error (20 percent coefficient of variation).

A total of 25 independent datasets were generated for each scenario. All datasets were generated by the authors through stochastic simulation from the model described above; no external or public data were used. Because the study evaluates diagnostic behaviour under known, induced influence mechanisms rather than predictive performance, it involves no training/test partition: each simulated dataset is analysed in full by the jackknife procedure.

### 2.3. Simulation Scenarios

Four distinct scenarios were defined:

• Scenario A (reference): baseline simulation without structural perturbation.
• Scenario B (outlier): one randomly selected individual had three observations multiplied by a factor of 5, generating extreme residual values.
• Scenario C (influential individual): one randomly selected individual was assigned a richer sampling design with additional time points, increasing its statistical leverage without introducing outliers.
• Scenario D (subpopulation): ten percent of individuals were randomly selected, and their observed concentrations were reduced by fifty percent, mimicking an unmodeled subpopulation.

**Figure 2:**
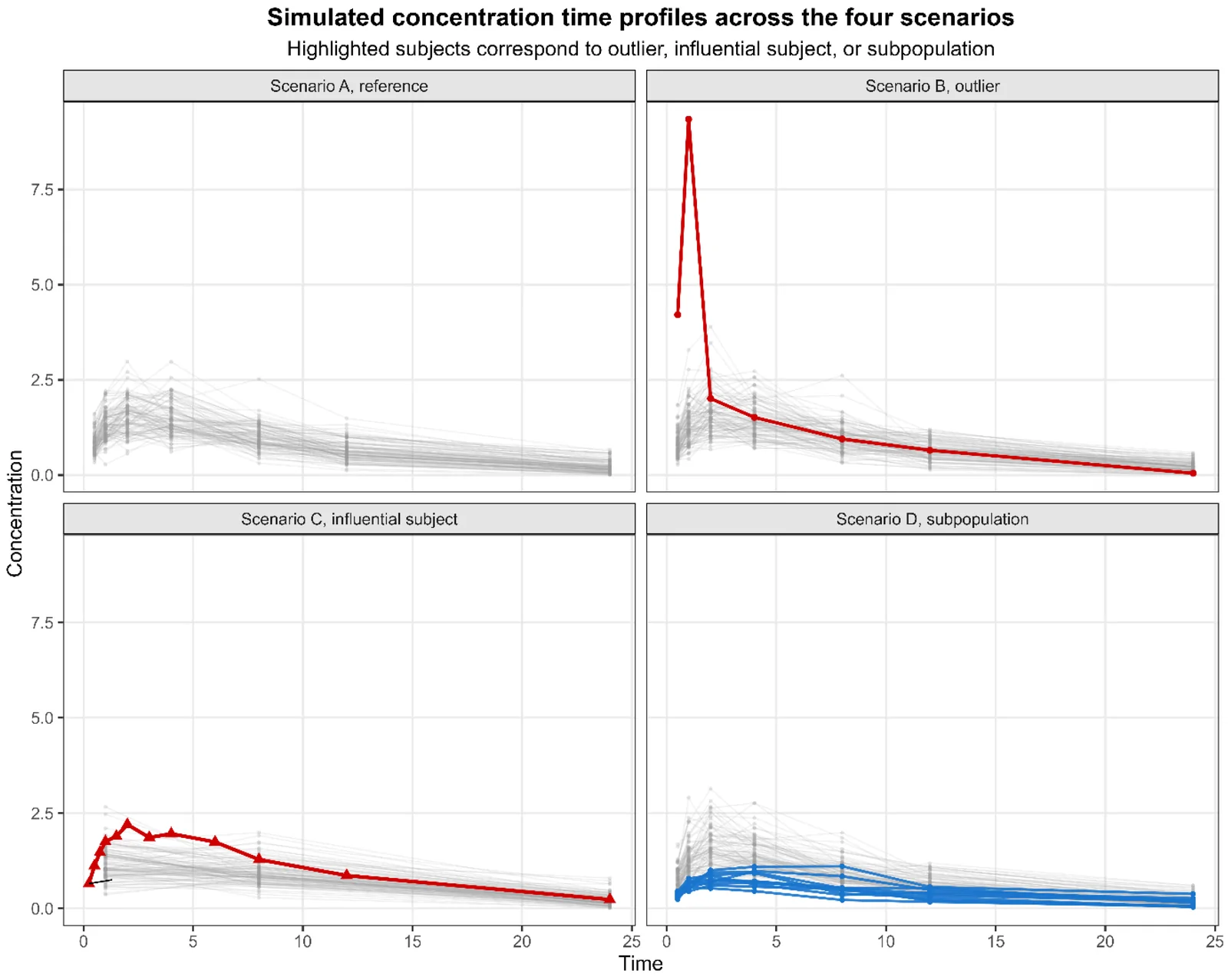
Simulated concentration–time profiles across scenarios. Simulated individual concentration–time profiles for the four scenarios. Highlighted profiles correspond to the manipulated individual in scenario B, the structurally influential individual in scenario C, and representative individuals from the latent subpopulation in scenario D. Scenario A represents the reference setting without induced influence.

Representative simulated concentration–time profiles for the four scenarios are shown in Figure 2.

### 2.4. Model Estimation

For each dataset, a population pharmacokinetic model was fitted using NONMEM. The structural model corresponded to the data-generating model. Parameter estimation was performed using the FOCEI algorithm.

### 2.5. Jackknife Procedure

For each dataset, a leave-one-out jackknife procedure was implemented. For a dataset with N individuals, N additional datasets were created, each excluding one individual.

Each jackknife dataset was fitted using the same model as the reference dataset. For each run, the objective function value and fixed-effect parameters were extracted.

### 2.6. Analysis

For each individual, the influence was quantified by comparing the parameter estimates and objective function value obtained from the jackknife dataset to those from the full dataset. Differences in objective function value (ΔOFV) and parameter estimates (Δθ) were computed. For each scenario, we also summarised the proportion of deletions producing a large likelihood change (|ΔOFV| ≥ 3.84; the χ^2^ critical value for one degree of freedom at α = 0.05, used here as a conventional likelihood-ratio reference rather than as a formal significance threshold for single-subject deletion), the proportion with a relative parameter shift above 1%, and the proportion of runs showing numerical instability (a change in condition number above 10%). Because the χ^2^(1) reference does not correspond to the null distribution of single-subject leave-one-out ΔOFV, we additionally derived an empirical screening threshold from the reference scenario (Scenario A), which contains no induced influence and therefore serves as an empirical null: the 95th percentile of its |ΔOFV| distribution ([153]) provides a data-driven cut-off for flagging potentially influential subjects without requiring additional simulation. The 1% relative parameter shift and 10% condition-number change are reported as descriptive summaries of perturbation magnitude and numerical stability, not as validated decision rules. Scenario-level median statistics were summarised with non-parametric bootstrap 95% confidence intervals (5000 replicates), and exact binomial 95% confidence intervals were calculated for detection proportions.

All analyses were implemented in R with automated generation of datasets, NONMEM control streams, execution of model runs, and extraction of results. Analyses used R version [4.6.1] with the packages [dplyr, readr, purr, ggplot2, tibble]. Population models were fitted with NONMEM version 7.6. PsN was not used at any stage, consistent with the PsN-independent design of the pipeline.

The identity of manipulated individuals was not stored during simulation and was reconstructed a posteriori based on scenario-specific criteria.

The jackknife procedure involves refitting the model after removal of each individual subject; with datasets of approximately 100 individuals, this required on the order of 100 model fits per dataset.

## 3. Results

### 3.1. Overall Quality of Model Estimation and Jackknife Runs

Across all scenarios, a total of 10,000 jackknife runs were generated and analyzed. All runs were successfully completed and met predefined quality criteria, including successful minimization and covariance steps. No dataset had to be excluded from the analysis.

The proportion of analyzable runs was therefore 100% across all scenarios, ensuring a complete and unbiased comparison of influence diagnostics.

### 3.2. Global Influence Patterns Across Scenarios

**Figure 3:**
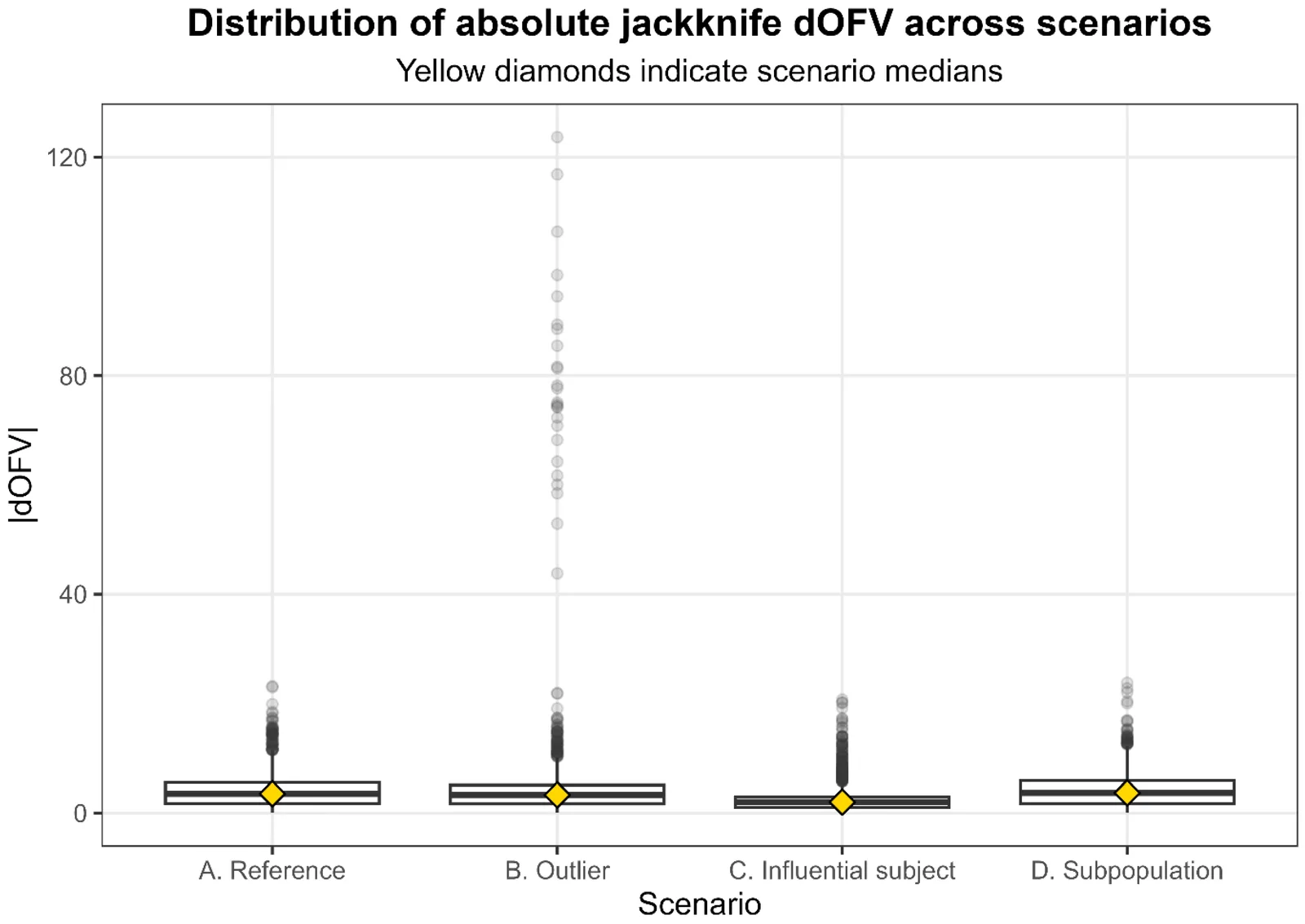
Distribution of absolute jackknife dOFV across scenarios. Boxplots and individual values of absolute changes in objective function value (|dOFV|) following individual deletion. Scenario A shows limited influence, whereas whereas scenarios B, C, and D exhibit broader distributions consistent with distinct mechanisms of influence.

The magnitude and distribution of individual influence varied markedly across scenarios (Table 1, Figure 3).

For each scenario, values summarise the distribution of the absolute change in objective function value (|ΔOFV|) and of the relative parameter perturbation (Δθ) across all leave-one-out deletions, together with the frequency of large likelihood changes, of substantial parameter shifts and of numerically unstable runs, and the within-dataset Spearman correlation between |ΔOFV| and Δθ. Medians are reported with interquartile range (IQR); the median Spearman ρ is reported with its 95% non-parametric bootstrap confidence interval (5000 replicates). “Large |ΔOFV|”, proportion of deletions with |ΔOFV| ≥ 3.84 (the χ^2^ critical value for 1 degree of freedom at α = 0.05); “Δθ > 1%”, proportion of deletions with a relative parameter shift above 1%; “Condition instability > 10%”, proportion of jackknife runs flagged for numerical instability (substantial change in condition number). ΔOFV, change in objective function value; Δθ, relative change in fixed-effect parameter estimates; CI, confidence interval; IQR, interquartile range.

In the reference scenario (A), the distribution of absolute changes in objective function value (|dOFV|) was narrow, reflecting the absence of influential individuals. Parameter perturbations were minimal, with no individual deletion inducing relative parameter changes greater than 5%.

In contrast, scenarios B, C, and D showed increased variability in |dOFV| and model stability. Scenario D exhibited the highest proportion of substantial changes in condition number, indicating increased numerical instability consistent with latent model misspecification. Scenario B showed moderate instability, while scenario C remained comparatively stable despite the presence of a highly informative individual.

**Figure 4:**
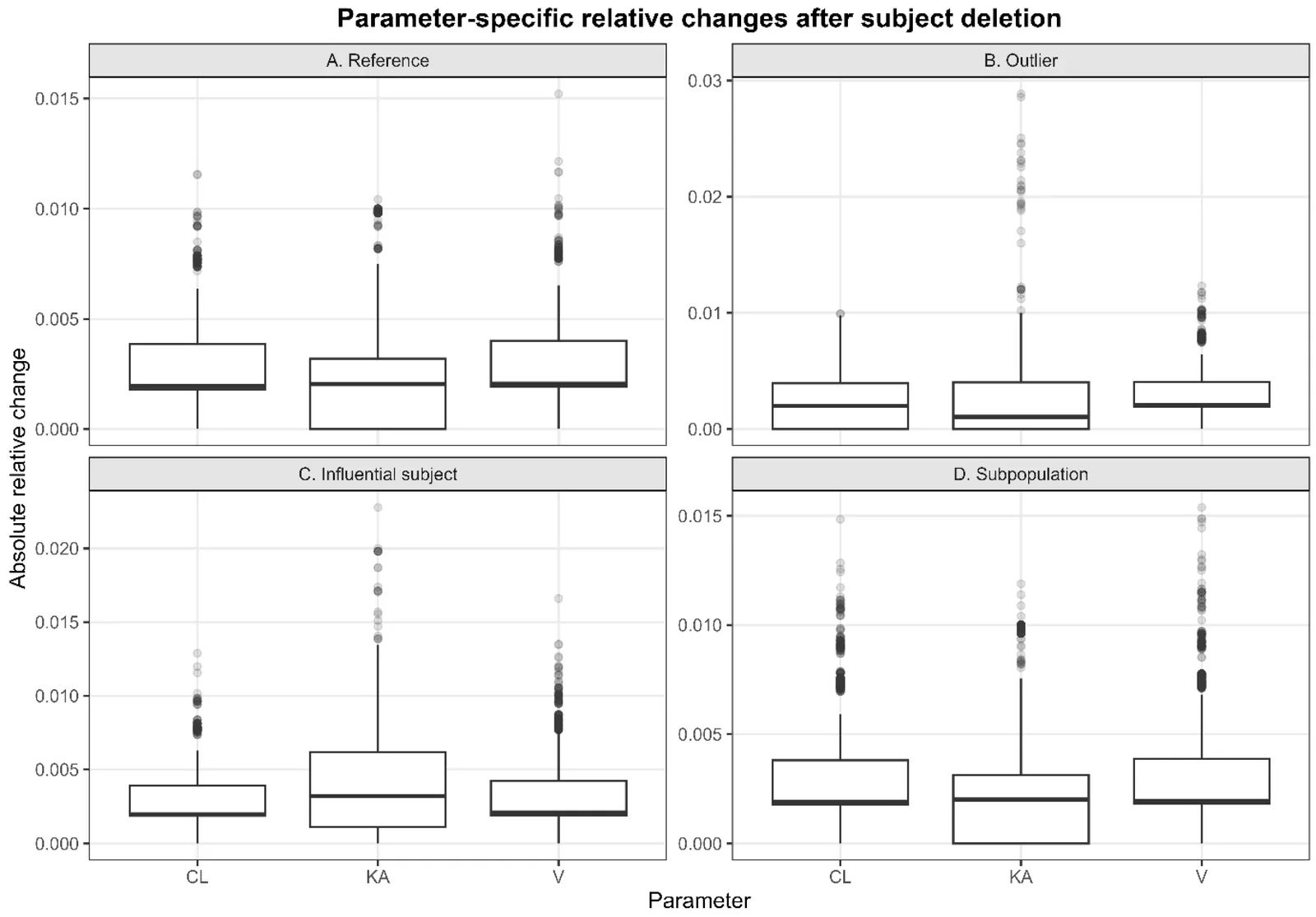
Parameter-specific perturbations following individual deletion.

Across all scenarios, relative parameter changes remained small overall, with no deletion exceeding 5% variation, indicating that substantial influence on parameter estimates can occur even in the absence of large relative parameter shifts (Figure 4).

Absolute relative changes in parameter estimates (CL, V, KA) after individual deletion, shown by scenario. Overall parameter perturbations remain limited across scenarios, even in the presence of substantial changes in objective function value.

### 3.3. Relationship Between ΔOFV and Parameter Perturbation

**Figure 5:**
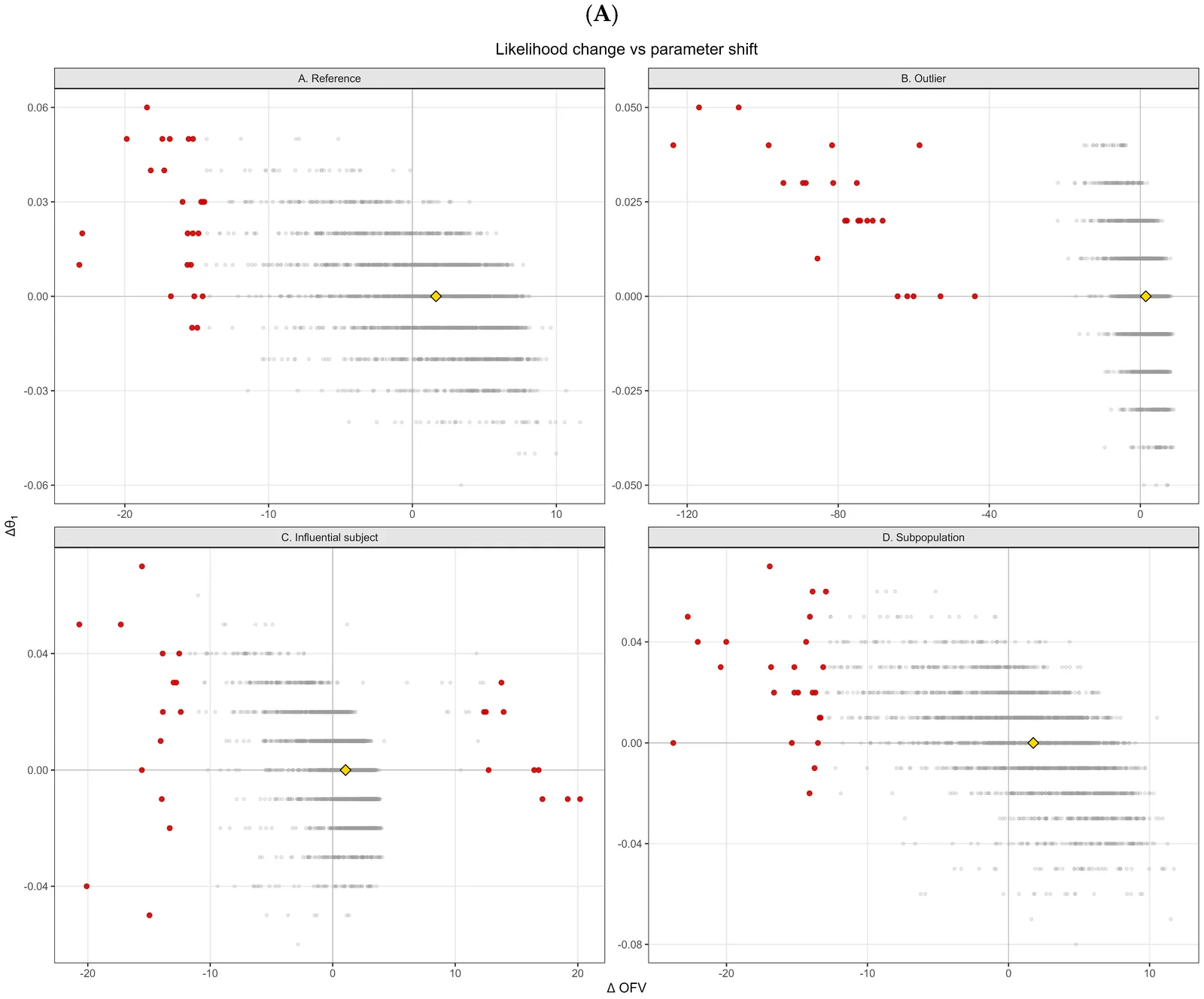

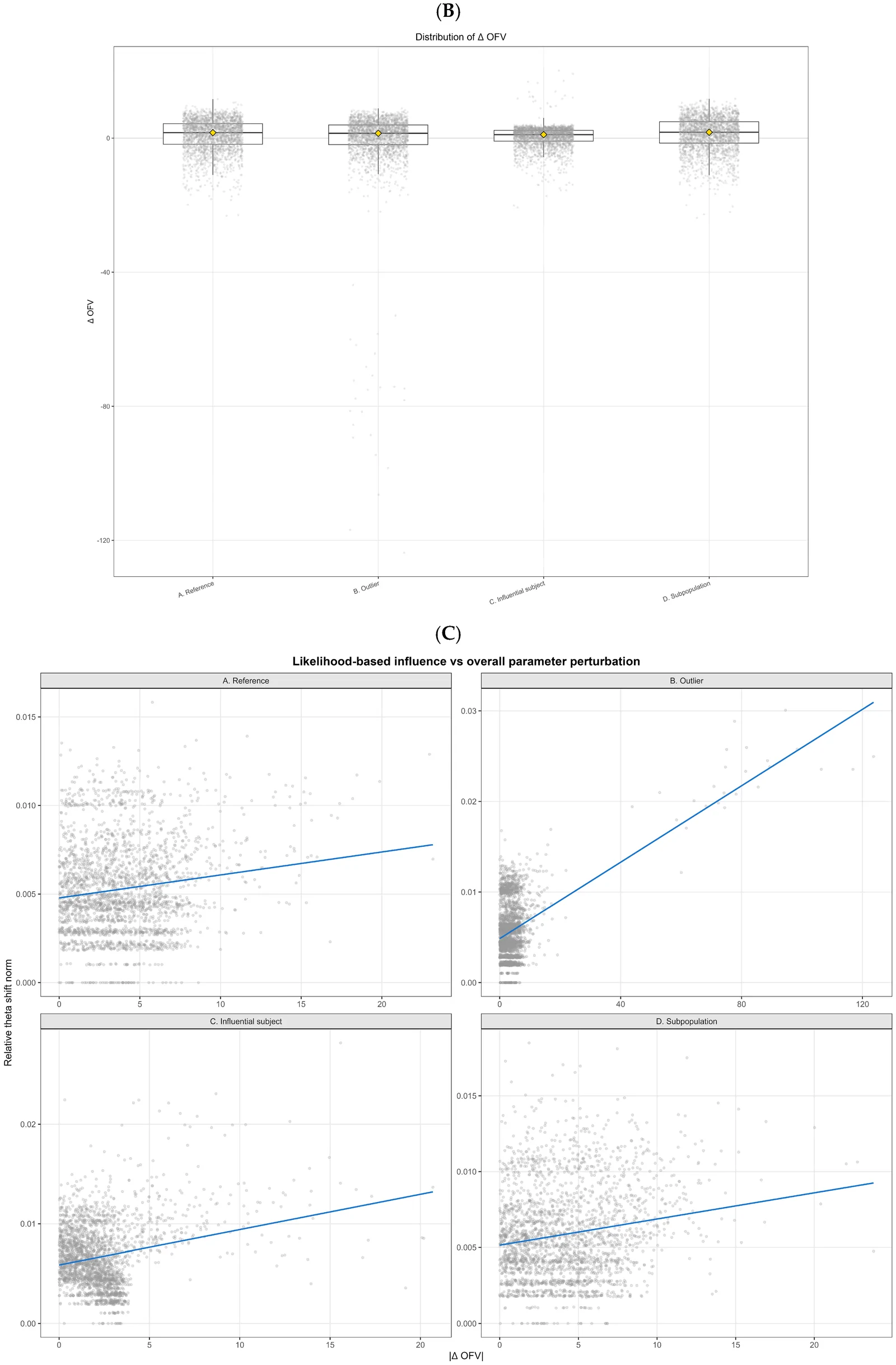
Relationship between likelihood-based influence and parameter perturbation. (**A**) ΔOFV versus Δθ1 across scenarios; red points denote extreme influence values and yellow diamonds denote scenario medians. (**B**) Distribution of ΔOFV across scenarios, with medians indicated. (**C**) Scatterplots of absolute ΔOFV versus the norm of the relative parameter change following individual deletion, shown by scenario, each point representing one individual. Together the panels show that large likelihood changes need not translate into large parameter shifts, and the absence of association in scenario C highlights the decoupling between influence on the likelihood and on parameter estimates.

The association between individual influence on the objective function and parameter estimates was generally weak (Table 1, Figure 5).

Median within-dataset Spearman correlations between |ΔOFV| and the relative parameter shift norm remained weak across all scenarios. Median correlations (95% bootstrap CI) were 0.05 (0.01–0.11) in scenario A, 0.15 (0.09–0.19) in scenario B, 0.13 (0.07–0.18) in scenario D, and −0.06 (−0.11 to 0.01) in scenario C, confirming an almost complete decoupling between likelihood changes and parameter perturbation in the structurally influential scenario.

This lack of association is illustrated in Figure 5, where scenario C shows no visible relationship between ΔOFV and parameter changes. Together, these results demonstrate that ΔOFV and Δθ capture distinct aspects of individual influence, and that parameter-based metrics alone did not reliably identify influential individuals across all scenarios.

### 3.4. Detection of Manipulated Individuals: Subject-Level Analysis

**Figure 6:**
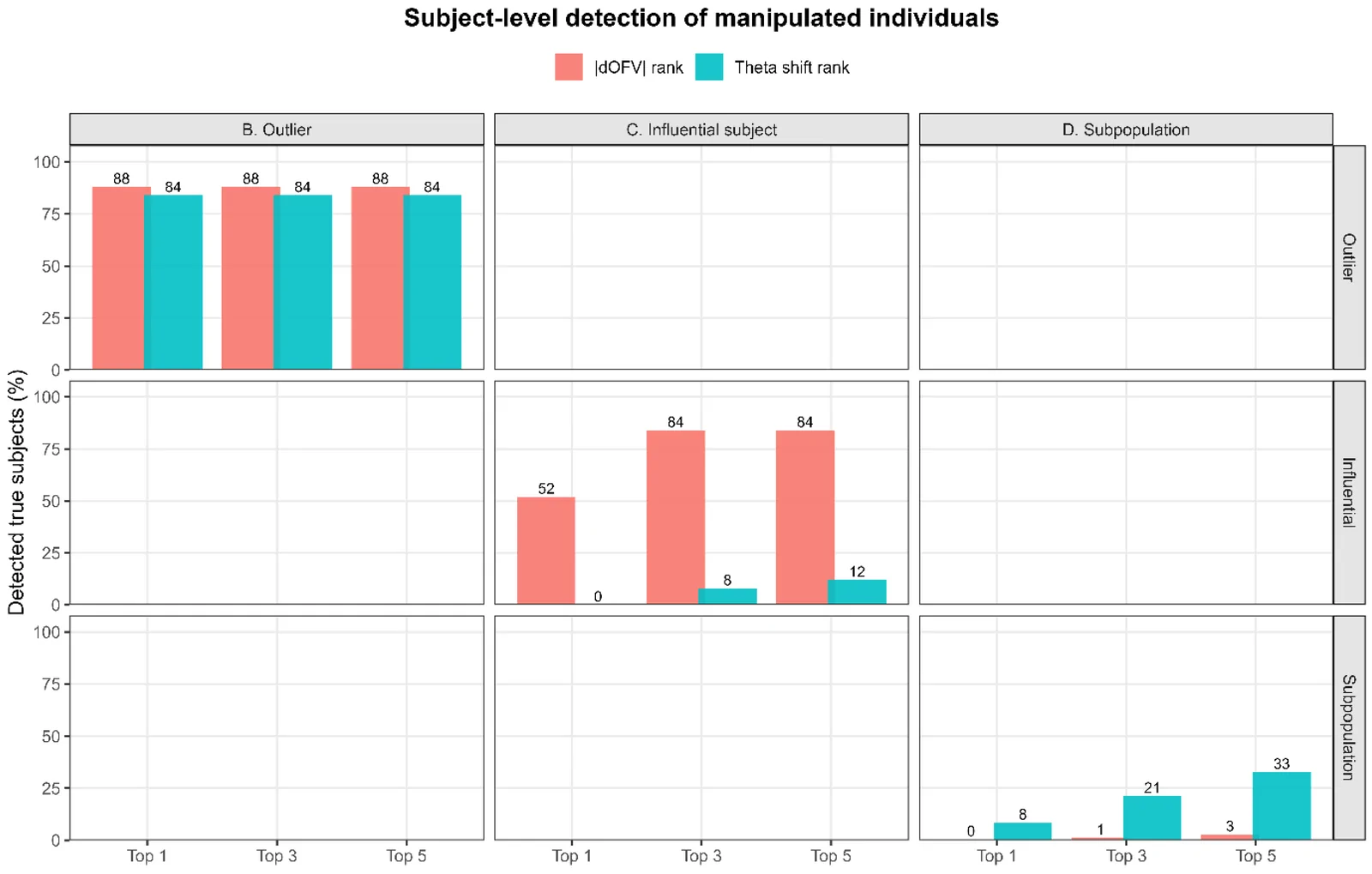
Subject-level detection of manipulated individuals.

The ability of jackknife-derived metrics to identify manipulated individuals differed across scenarios (Table 2, Figure 6).

Proportion of true manipulated individuals identified within the top 1, top 3 and top 5 ranks based on absolute ΔOFV and parameter-based metrics. ΔOFV consistently identifies structurally influential individuals, whereas parameter-based metrics do not identify the structurally influential individual in scenario C.

In scenario B (residual outliers), both |dOFV|-based and parameter-based rankings performed optimally. The manipulated individual was consistently ranked first, with a median rank of 1 for both metrics.

In scenario C (structurally influential individual), a marked divergence between metrics was observed. The influential individual was systematically identified by |dOFV|, with a median rank of 1. In contrast, parameter-based rankings failed to detect this individual, with a median rank of approximately 25, corresponding to near-random ordering among subjects.

In scenario D (latent subpopulation), subject-level detection was limited for both approaches. Median ranks were approximately 31 for |dOFV| and 10 for parameter-based metrics, indicating that no single individual consistently exhibited strong influence when considered in isolation.

### 3.5. Detection of Manipulated Individuals: Dataset-Level Analysis

**Figure 7:**
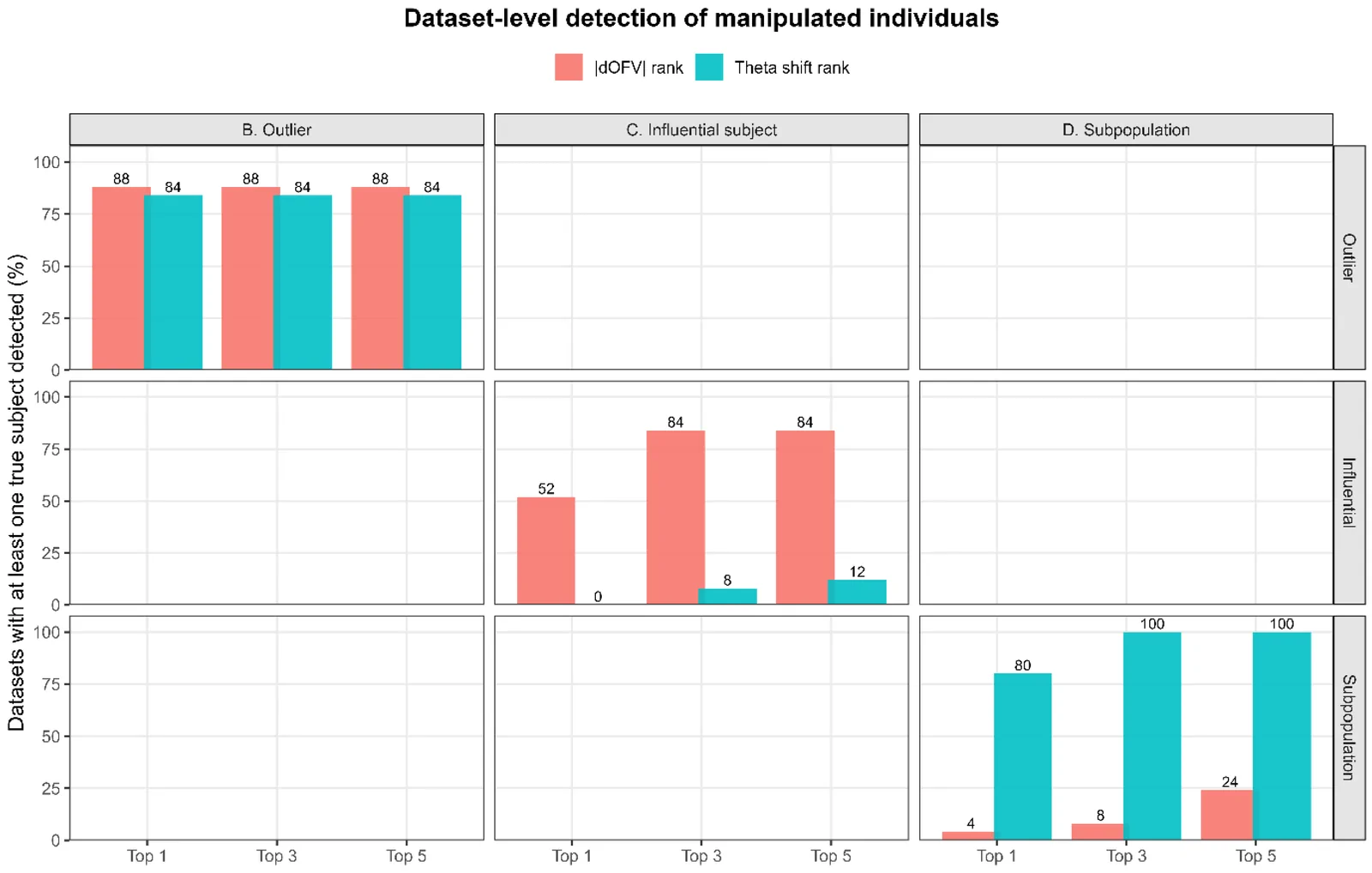
Dataset-level detection of manipulated individuals.

To account for multiple manipulated individuals in scenario D, detection performance was also evaluated at the dataset level (Table 2, Figure 7).

Proportion of datasets in which at least one true manipulated individual is identified within the top 1, top 3, top 5, and top 10 ranks. Detection remains effective in scenario D despite poor subject-level identification, reflecting the presence of a latent subpopulation.

In scenarios B and C, dataset-level results mirrored subject-level findings, with near-perfect identification of the manipulated individual. For scenario C, subject-level and dataset-level detection are identical by construction, as only one individual is manipulated per dataset.

In scenario D, although individual-level detection was poor, dataset-level detection remained effective. At least one individual belonging to the latent subpopulation was frequently but not systematically identified (Table 2) among the top-ranked subjects. Using parameter-based metrics, dataset-level detection reached 80% (95% CI, 59–93%) at Top 1 and 100% (95% CI, 86–100%) at both Top 3 and Top 5, indicating that the presence of a subpopulation could be reliably detected at the dataset level even when individual identification remained uncertain.

### 3.6. Summary of Findings Across Scenarios

Taken together, these results highlight three distinct patterns of influence.

Residual outliers (scenario B) were readily detected by all metrics. Structurally influential individuals (scenario C) were exclusively identified by ΔOFV, while parameter-based metrics did not capture this form of influence. Latent subpopulation effects (scenario D) were not detectable at the individual level but could be identified at the dataset level through aggregated influence signals.

These findings underscore that individual influence is mechanism-dependent and that no single metric dominates across all mechanisms: ΔOFV is the most reliable metric for detecting single-subject influence, including the structurally informative case, whereas parameter-based metrics are more sensitive to latent subpopulations at the dataset level.

## 4. Discussion

### 4.1. Principal Findings

This study evaluates individual influence in nonlinear mixed-effects models across distinct mechanistic scenarios. The picture that emerges is that detectability depends on how the influence arises. Residual outliers were the easy case, flagged by every diagnostic because model misfit, parameter perturbation and individual influence rise together. A structurally influential subject was harder: its increased informational content produced a marked change in ΔOFV while leaving the parameter estimates (and therefore parameter-based diagnostics) largely unchanged. A latent subpopulation was the most elusive, escaping detection at the individual level yet becoming visible once influence was aggregated across the dataset (Figure 6 and Figure 7, Table 2). The ΔOFV was the most consistent marker across these mechanisms, although it should be regarded as complementary to parameter-based measures rather than a replacement for them.

### 4.2. ΔOFV as a Direct Measure of Influence

The leave-one-out jackknife approach provides a direct estimate of the impact of each individual on model estimation. By recomputing the objective function after removing each subject, ΔOFV quantifies the contribution of that individual to the likelihood of the model, without relying on approximations derived from the full fit.

This contrasts with individual objective function contributions (iOFV), which are computed conditional on the full dataset and therefore do not reflect how parameter estimates would change if an individual were removed [6]. Similarly, residual-based diagnostics such as conditional weighted residuals (CWRES) assess local model fit but do not capture global influence on parameter estimation [5].

Our results confirm that ΔOFV remains informative across all scenarios, including situations in which residual- or parameter-based diagnostics provide only partial information. This supports ΔOFV as the most direct measure of individual influence on model estimation. This interpretation is consistent with the case-deletion literature, in which exact and approximate ΔOFV methods have been developed specifically to quantify the influence of individual data on the model fit [8,9,11], and it complements shrinkage-aware critiques of conditional diagnostics [7], which caution against using full-fit quantities to judge how much an individual actually affects estimation.

### 4.3. Decoupling Between ΔOFV and Parameter Changes

A key result of this study is the weak association between ΔOFV and parameter perturbations across most scenarios, and particularly the near-complete decoupling observed in scenario C (Figure 5, Table 1).

In this scenario, a single individual with a richer sampling design exerted a strong influence on the objective function yet had minimal impact on parameter estimates. This illustrates that influence on model likelihood and influence on parameter values are distinct concepts. An individual may strongly constrain the likelihood surface without substantially shifting the estimated parameters.

This distinction also matters in practice. Parameter-based metrics, such as Δθ or relative parameter changes, describe the sensitivity of parameter estimates to the removal of an individual. Although this information is valuable, it should not be interpreted as a direct measure of individual influence on model estimation. Our results show that these two concepts may diverge substantially when an individual’s contribution is primarily informational rather than structural. The absence of a clear association between |dOFV| and overall parameter perturbation in scenario C directly supports this interpretation (Figure 5).

The narrow bootstrap confidence intervals around the scenario-specific median correlations further support that this weak association reflects a robust property of the simulated mechanisms rather than sampling variability.

### 4.4. Limitations of Residual-Based and Parameter-Based Diagnostics

Residual-based diagnostics are widely used in pharmacometrics due to their simplicity and availability. CWRES, in particular, are commonly used to identify model misspecification and outliers [3]. However, their interpretation is inherently local, as they quantify the discrepancy between observed and predicted values for each individual. Consequently, CWRES quantify model fit rather than the influence of an individual on the estimation of population parameters.

In our study, CWRES-based reasoning would correctly identify residual outliers (scenario B) but would fail to detect structurally influential individuals (scenario C), as these individuals do not exhibit large residuals. This limitation has been previously discussed in the context of shrinkage and diagnostic reliability [4,7].

Similarly, parameter-based diagnostics quantify the extent to which parameter estimates change after deletion of an individual. Although closely related in some situations, parameter perturbation and individual influence describe different properties of model behaviour. While this is true for certain types of influence, such as outliers or structural misspecification, it does not hold for all mechanisms. Our results demonstrate that parameter changes can remain small even when the contribution to the likelihood is substantial, and that parameter-based rankings may therefore miss highly influential subjects (Figure 6, Table 2).

### 4.5. Detection of Latent Subpopulations

Scenario D highlights a different type of challenge, where influence arises from a latent subpopulation rather than a single individual. In this case, neither ΔOFV nor parameter-based metrics were effective at identifying individual subjects belonging to the subpopulation (Figure 6, Table 2).

However, detection became possible when considering the dataset as a whole. The presence of at least one subpopulation member among the top-ranked individuals was frequently observed, indicating that aggregated influence signals can reveal hidden heterogeneity even when individual identification is uncertain (Figure 7, Table 2).

This finding is consistent with previous work showing that unmodelled subpopulations can lead to increased variability, instability, and multimodality in model estimation [5,6]. It also suggests that influence diagnostics should be interpreted at multiple levels, including both individual and dataset perspectives.

### 4.6. Practical Implications for Pharmacometric Workflows

For routine pharmacometric analyses, these results suggest a hierarchical approach to influence assessment.

Residual-based diagnostics and iOFV contributions can be used as efficient initial screening tools because they identify model misfit at minimal computational cost. However, these diagnostics should be viewed as complementary to, rather than substitutes for, direct influence assessment based on jackknife deletion.

When influential individuals are suspected, jackknife deletion provides a definitive assessment. Although computationally intensive, its implementation can be automated, as demonstrated in this study, and does not require specialized tools such as PsN.

The distinction between influence on likelihood and influence on parameters should be explicitly considered when interpreting results. In particular, reliance on parameter changes alone may lead to under-detection of influential individuals in scenarios where information content plays a dominant role.

### 4.7. Limitations and Future Directions

Several limitations should be kept in mind. The computational cost of the jackknife scales linearly with the number of subjects, which may limit its routine application in large datasets.

Our simulations rest on a single one-compartment model fitted with FOCEI and on one perturbation intensity per scenario; how far the qualitative separation between ΔOFV and Δθ extends to richer settings (multi-compartment or nonlinear-elimination models, a range of perturbation intensities, and gradient effects) remains to be tested. We see this systematic evaluation as the natural continuation of the work. A second limitation is that the identity of the manipulated subjects was not recorded during simulation and had to be reconstructed afterwards: exactly, from the sampling design, for Scenario C, but only approximately, from normalized concentration profiles, for Scenario D. Any misclassification of subpopulation membership would tend to lower the Scenario D detection rates, which are therefore best read as conservative. Finally, we quantified influence on the fixed-effect parameters alone; extending the jackknife to the variance components of the random effects (the Ω matrix) is a further worthwhile direction.

## 5. Conclusions

Whether a subject matters to a population model depends on how its influence arises, which is why no single diagnostic is sufficient. In our simulations, leave-one-out ΔOFV identified influential subjects across every mechanism examined, including the information-rich subject of Scenario C, whose imprint on the likelihood was largely invisible to parameter-based measures. The parameter perturbation (Δθ) captured a different quantity (how far the estimates themselves move when a subject is removed) and was the more sensitive marker of a latent subpopulation once influence was assessed at the dataset level. The two measures are therefore complementary: The ΔOFV reflects how strongly a subject shapes the fit, Δθ how much it shifts the parameters, and neither alone is sufficient.

In practice, this favours matching the diagnostic to the question and treating inexpensive screening tools such as CWRES and iOFV as a first pass rather than a definitive assessment; where influence remains in doubt, jackknife deletion resolves it directly. By making that procedure available without PsN and on standard Windows systems, the pipeline described here should make case-deletion diagnostics more accessible for routine pharmacometric practice.

## 6. Key Points

• Individual influence in nonlinear mixed-effects models is mechanism-dependent and cannot be fully characterized by a single diagnostic.
• Jackknife-based ΔOFV provides a robust and general measure of influence, capturing both residual outliers and structurally influential individuals.
• Parameter-based metrics quantify parameter sensitivity and may not identify all influential individuals.
• Residual-based diagnostics are effective for detecting local model misfit but do not reflect global influence on model estimation.
• Latent subpopulation effects are difficult to identify at the individual level but can be detected at the dataset level through aggregated influence signals.

## Figures and Tables

**Table 1:** Scenario-level summary of jackknife influence metrics.

Scenario	Median |ΔOFV| [IQR]	Median Relative Δθ [IQR]	Large |ΔOFV| (%)	Δθ > 1% (%)	Condition Instability > 10% (%)	Median Spearman ρ (95% CI)
A. Reference	3.50 [1.71, 5.59]	0.0048 [0.0034, 0.0068]	45.2	1.0	44	0.05 (0.01 to 0.11)
B. Outlier	3.29 [1.67, 5.10]	0.0049 [0.0031, 0.0074]	41.4	6.3	64	0.15 (0.09 to 0.19)
C. Influential subject	1.99 [1.01, 2.91]	0.0063 [0.0044, 0.0086]	10.4	6.7	24	−0.06 (−0.11 to 0.01)
D. Subpopulation	3.67 [1.68, 5.94]	0.0054 [0.0037, 0.0075]	48.0	6.4	76	0.13 (0.07 to 0.18)

**Table 2:** Detection performance of jackknife-derived influence metrics.

Scenario	Detection Level	Truth Type	Metric	Top 1	Top 3	Top 5	Top 10	Median Rank
B. Outlier	Subject level	outlier	|ΔOFV| rank	88%	88%	88%	92%	1.0
B. Outlier	Subject level	outlier	Δθ rank	84%	84%	84%	88%	1.0
B. Outlier	Dataset level	outlier	|ΔOFV| rank	88%	88%	88%	92%	1.0
B. Outlier	Dataset level	outlier	Δθ rank	84%	84%	84%	88%	1.0
C. Influential subject	Subject level	influential	|ΔOFV| rank	52%	84%	84%	92%	1.0
C. Influential subject	Subject level	influential	Δθ rank	0%	8%	12%	28%	25.0
C. Influential subject	Dataset level	influential	|ΔOFV| rank	52%	84%	84%	92%	1.0
C. Influential subject	Dataset level	influential	Δθ rank	0%	8%	12%	28%	25.0
D. Subpopulation	Subject level	subpopulation	|ΔOFV| rank	0.4%	1.2%	2.8%	13.6%	31.0
D. Subpopulation	Subject level	subpopulation	Δθ rank	8.4%	21.2%	32.8%	53.2%	10.0
D. Subpopulation	Dataset level	subpopulation	|ΔOFV| rank	4%	8%	24%	64%	9.0
D. Subpopulation	Dataset level	subpopulation	Δθ rank	80%	100%	100%	100%	1.0

Subject-level detection indicates the proportion of true manipulated subjects identified within the top-ranked positions. Dataset-level detection indicates the proportion of datasets in which at least one true manipulated subject was identified within the top-ranked positions. Values are percentages; median rank refers to the rank of the manipulated subject(s) under each metric. |ΔOFV|, absolute change in objective function value; Δθ, relative parameter shift.

## Data Availability

All data analysed in this study were generated by computer simulation. The R script implementing the PsN-independent automated jackknife pipeline and the post-processing analyses is openly available on Zenodo at https://doi.org/10.5281/zenodo.21772026.
